# The Micronizer: A Novel Sharp-Bladed Device for Fat Graft Micronization to Optimize Adipocyte Viability

**DOI:** 10.7759/cureus.113683

**Published:** 2026-07-30

**Authors:** Oscar A Vazquez, Nicholas De Leo, Hilton Becker

**Affiliations:** 1 Surgery, University of Florida, Gainesville, USA; 2 Plastic and Reconstructive Surgery, Cleveland Clinic Florida, Weston, USA; 3 Surgery, Florida Atlantic University, Boca Raton, USA

**Keywords:** adipocyte viability, body contouring, fat grafting, lipoaspiration, stromal vascular fraction

## Abstract

Reducing fat particle size before injection is anticipated to promote adipocyte survival and facilitate injection through smaller cannulas or needles. We present the Micronizer (Marina Medical, Davie, FL), a Luer-to-Luer connector housing an ultrasharp, double-sided blade that reduces fat particle size through direct sharp dissection rather than mechanical emulsification. Through its dual-configuration design, comprising 2.4- and 1.2-mm apertures, the Micronizer cuts directly along the axis of fat passage, enabling stepwise reduction in particle size. Fat harvested using a 2.5-3.0-mm cannula is processed by repeated passage between syringes through the device. The direct sharp-cutting mechanism is anticipated to reduce fat particle size while limiting compressive forces, with the goal of preserving graft viability. We describe the complete processing protocol. The Micronizer offers a standardized, reproducible method for fat graft micronization that achieves particle sizes anticipated to be optimal for vascularization. Potential applications include revision breast reconstruction, primary breast reconstruction, breast augmentation, and facial rejuvenation. Comparative studies evaluating cell viability and long-term volume retention are warranted to validate the anticipated advantages of direct sharp-blade processing over conventional compressive techniques.

## Introduction

Autologous fat grafting has become one of the most commonly performed procedures in both reconstructive and aesthetic plastic surgery, with applications ranging from breast reconstruction, breast augmentation, and facial rejuvenation to numerous other regenerative procedures [1]. Despite its widespread adoption, variable graft survival rates, reported to range between 25% and 80%, remain a significant limitation [1]. Standardized techniques for harvesting, processing, and injecting fat grafts were first outlined by Coleman SR over 30 years ago [2]. Subsequent work by Eto H et al. defined three zones within a fat graft, laying the groundwork for determining the optimal fat graft size, and demonstrated that only peripheral adipocytes within 300 μm of the graft surface survive through direct diffusion [3]. According to this model, necrosis and regeneration then occur over a further 600 μm as revascularization develops. Fat particles measuring 0.5-1.0 mm in diameter therefore represent an optimal size range.

This understanding has driven interest in reducing fat particle size to optimize the surface-area-to-volume ratio and enhance graft vascularization. Eto H et al. demonstrated that smaller fat particles exhibit improved survival because of reduced central necrosis [3]. Surgeons often lack specific information regarding the diameter of the harvesting cannula, the size of the harvested fat particles, and their relationship to the cannula or needle used for injection. Studies have demonstrated that fat particles containing mature adipocytes are especially fragile and susceptible to mechanical trauma. When fat is forcefully injected through a cannula or needle with an inadequately small internal diameter, the adipocytes may become damaged, leading to cell death [4]. Therefore, reducing the size of fat particles when necessary before injection is important to promote adipocyte survival and facilitate injection through smaller cannulas or needles.

Tonnard P et al. introduced nanofat grafting, a technique in which fat is emulsified by repeated passage through progressively smaller Luer-to-Luer connectors, destroying viable adipocytes while preserving adipose-derived stem cells (ASCs) for regenerative applications [5]. Although nanofat has demonstrated utility in skin rejuvenation, its indications are limited to skin regeneration and do not encompass volume restoration because the preparation does not contain viable adipocytes [5]. A recent expert consensus recommended that nanofat be defined as lipoaspirate that undergoes washing, emulsification through a 1.2-1.6-mm connector for 20-30 passes, and a final filtration step [6]. The term “nanofat” may therefore be misleading, as the preparation functions primarily as a regenerative rather than a volumetric graft.

The mechanical processing landscape has expanded considerably in recent years. Ma X et al. conducted a scoping review of 89 studies on mechanically micronized adipose-derived products, categorizing the techniques into three primary methods: shuffling-based emulsification, rotating blade systems, and filtration bag systems. Within this taxonomy, blade-based systems represent a distinct approach that employs sharp dissection rather than compressive forces to reduce particle size [7].

The Micronizer (Marina Medical, Davie, FL) is a patented Luer-to-Luer connector that employs a sharp, double-edged blade to reduce fat particle size through direct sharp dissection rather than compression-based mechanical emulsification. The initial version consisted of a No. 15 blade housed within a Luer-to-Luer connector, which assisted in reducing fat particles for injection through needles [4]. Compared with other described blade-equipped devices, we hypothesize that the Micronizer differs in two key respects. First, the Micronizer’s blade is aligned directly along the trajectory of fat passage, producing a direct cut, whereas the Adinizer blade is positioned at 90° to the direction of fat movement, resulting in an indirect cut. A direct-cutting mechanism is anticipated to reduce lateral shearing forces and minimize adipocyte trauma compared with an indirect approach. Second, the Micronizer is available in two configurations, with 2.4- and 1.2-mm apertures, enabling stepwise particle size reduction from macrofat through microfat to nanofat within a single processing workflow. By employing direct sharp-blade technology rather than compressive forces, the Micronizer cuts fat particles into defined sizes and is anticipated to minimize cellular trauma. This technical report describes the device and outlines the processing technique.

## Technical report

Device description

**Figure 1:**
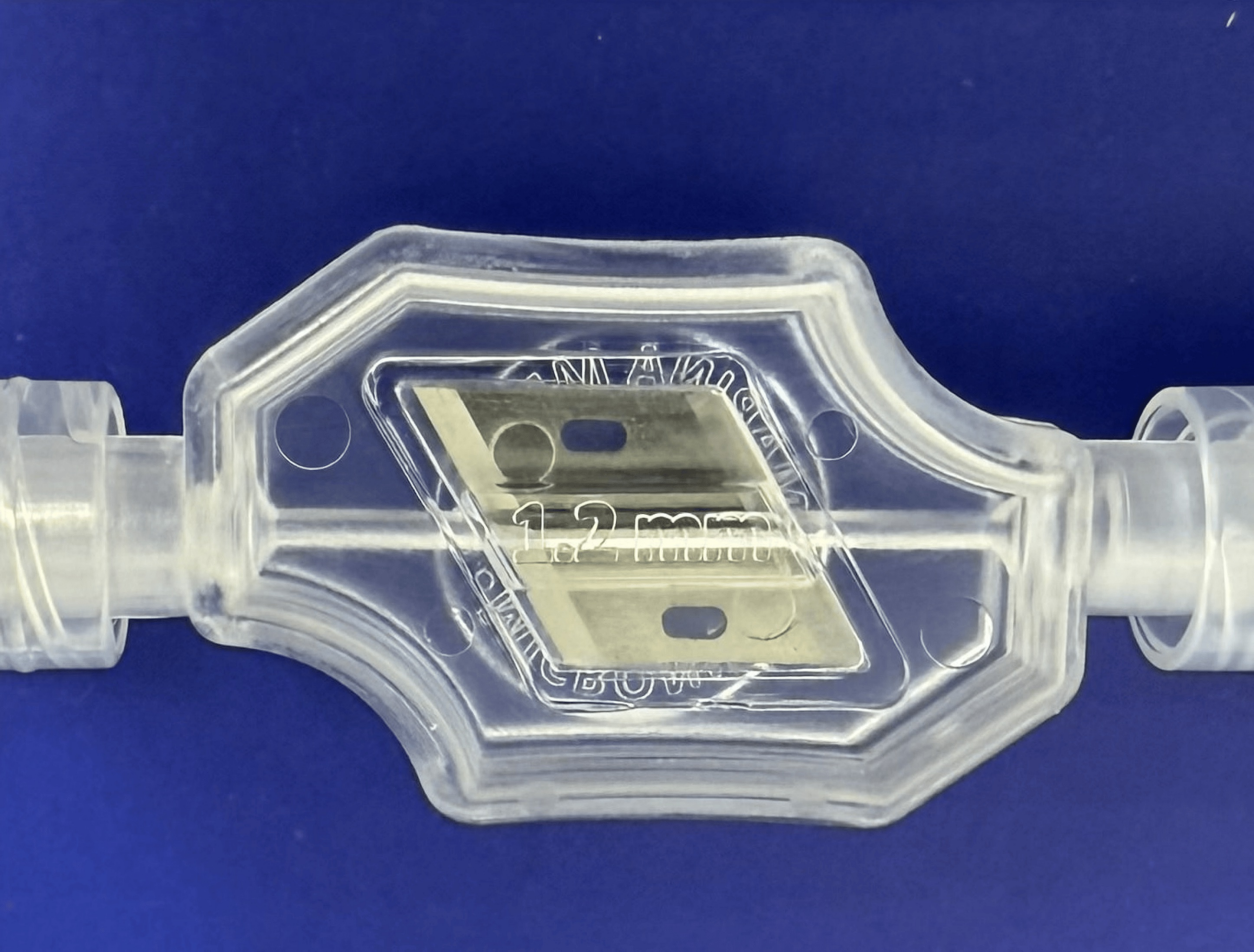
The Micronizer device. A transparent Luer-to-Luer connector houses an ultrasharp, double-sided blade and has a 1.2-mm aperture for secondary processing. The blade is aligned along the axis of fat passage, producing a direct cut.

The Micronizer consists of a transparent polycarbonate Luer-to-Luer connector housing an ultrasharp, double-sided stainless-steel blade (Figure 1). Two configurations are available: a device with a 2.4-mm internal diameter for initial processing and a device with a 1.2-mm internal diameter for further size reduction. The blade is oriented along the axis of fat passage, allowing the fat to be sharply divided as it passes through the aperture rather than being compressed through a restrictive opening or cut perpendicular to its trajectory.

Fat harvesting

Fat is harvested using a standard tumescent technique with a 2.5- or 3.0-mm multihole cannula connected to a 10- or 20-mL syringe or a canister under low negative pressure.

Processing protocol

The processing protocol consists of five steps:

Initial Preparation 

Harvested fat is allowed to separate by gravity, centrifugation, or straining. The fat is then strained and rinsed with normal saline before being transferred to either a 10- or 20-mL syringe. The infranatant fluid and oil supernatant are removed.

Primary Micronization 

**Figure 2:**
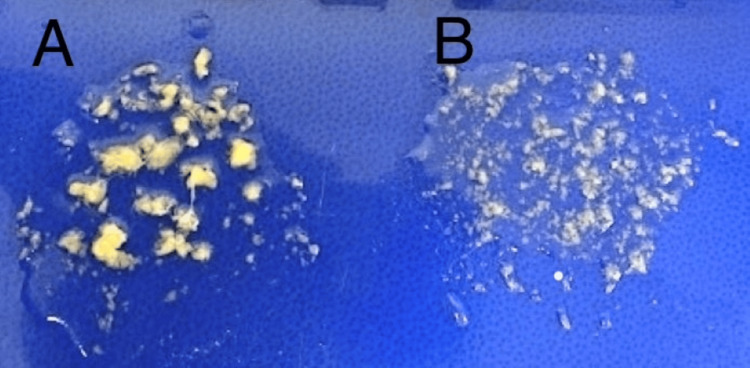
Fat particle-size reduction using the 2.4-mm Micronizer. Harvested fat particles before processing (A) are reduced to an estimated diameter of approximately 1.0 mm after processing (B). Both samples were photographed simultaneously under identical conditions. For a calibrated particle-size reference, see Figure 3.

The 2.4-mm Micronizer is connected between two appropriately sized syringes. With a single pass through the device, the particle size is reduced to approximately 1.0 mm, making the fat suitable for injection through a 1.0-mm cannula or a 16-gauge needle (Figure 2). If the surgeon requires smaller particles for injection through a narrower cannula or needle, additional passes can further reduce the particle size. As the number of passes through the Micronizer increases, more oil is released from the adipocytes, resulting in a higher relative concentration of stromal vascular fraction (SVF)-rich fat. The released oil should be removed by straining or centrifugation.

Secondary Micronization (Optional)

**Figure 3:**
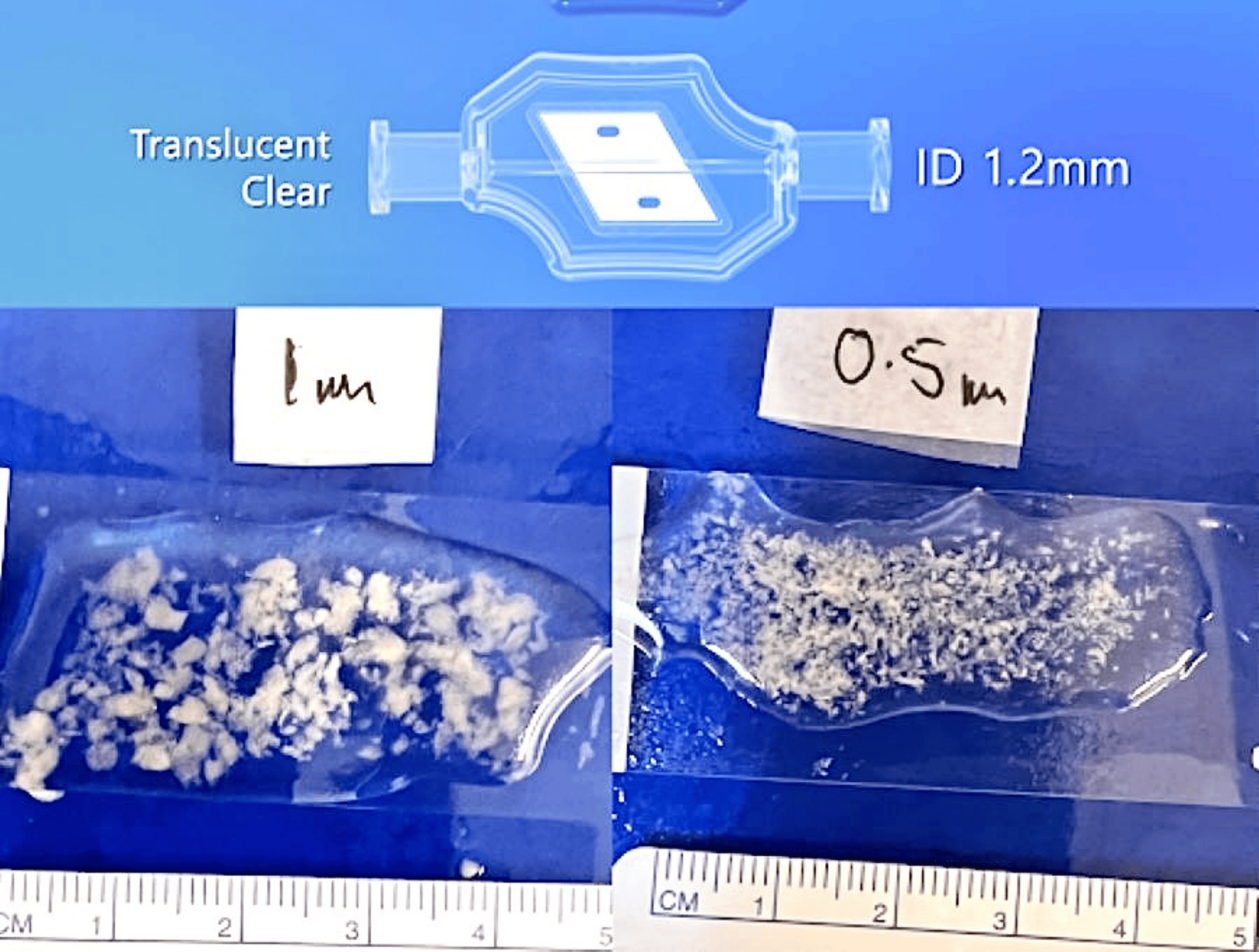
Secondary processing with the 1.2-mm Micronizer. Fat particles measuring approximately 1.0 mm before processing (left) are reduced to an estimated diameter of approximately 0.5 mm after processing (right), making them suitable for injection through a 22-gauge needle.

For applications requiring smaller particles, the 1.0-mm fat particles are processed through the 1.2-mm Micronizer, yielding particles of approximately 0.5 mm (Figure 3). The number of passes can be adjusted based on the desired particle size and intended injection device, allowing the surgeon to customize processing for the specific clinical application. These smaller fat particles can be effectively injected using 22- to 25-gauge needles.

Tertiary Micronization (Optional)

Additional passes through the 1.2-mm device produce particles of approximately 0.25 mm, which are suitable for fine-needle injection.

Final Processing

**Figure 4:**
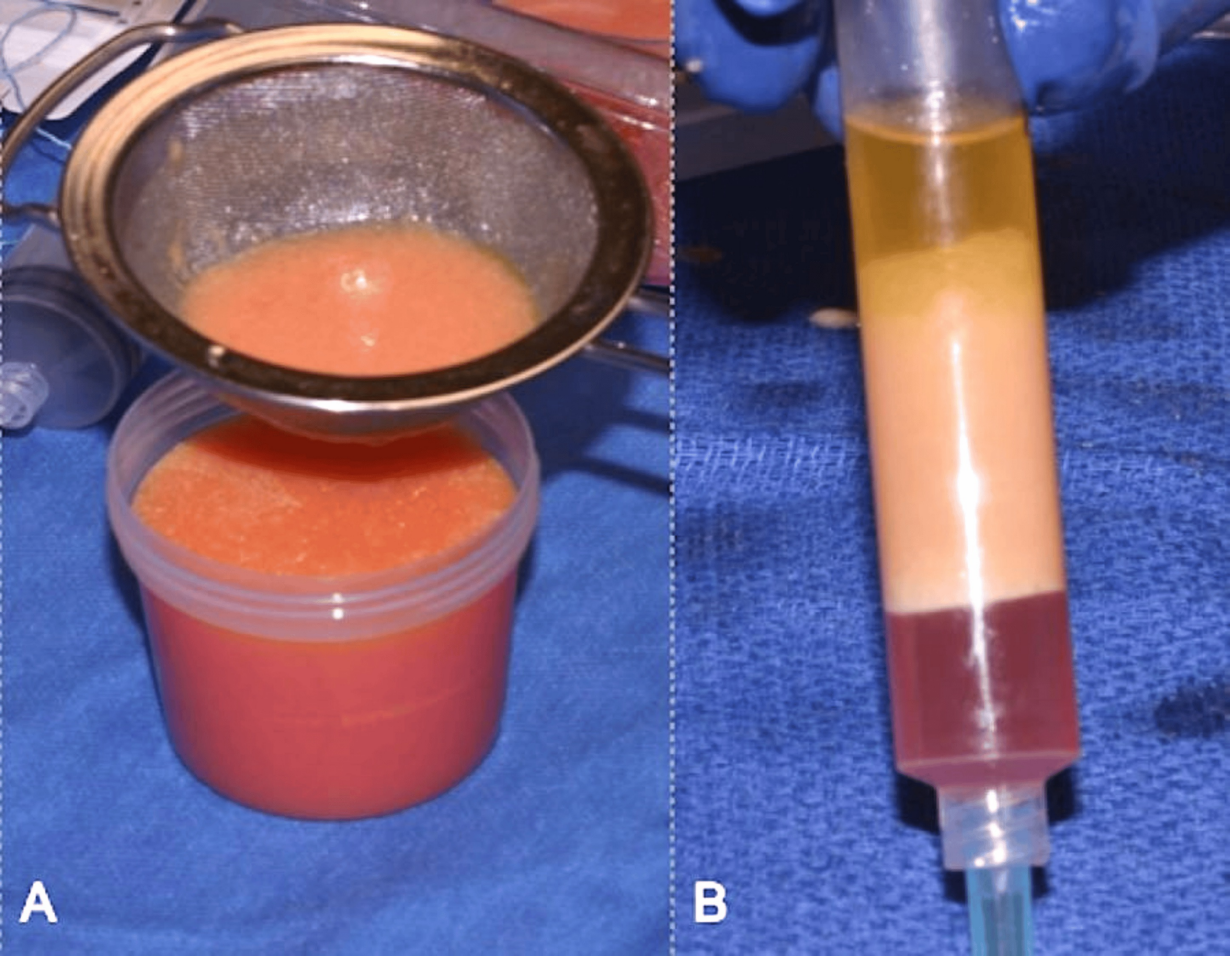
Fat-processing steps. (A) Straining of the micronized fat emulsion to remove connective-tissue strands. (B) Preparation after centrifugation showing separation into an oil layer (top), concentrated ultra-micro fat (middle), and an aqueous fraction (bottom).

Micronized fat undergoes straining or centrifugation to produce nanofat (Figures 4A-4B). During centrifugation, the oil is discarded, resulting in a more concentrated preparation of ultra-micro fat and SVF containing stem cells.

To obtain nanofat, the fat is further micronized to achieve a particle size that can be injected through a 25-gauge needle. Straining the fat makes it possible to inject it through a 27-gauge needle.

The particle sizes reported in this study were estimated through gross visual comparison with a calibrated ruler, as demonstrated in Figure 3. Formal microscopic particle-size analysis was not performed; this represents an area for future investigation.

Particle size and injection cannula correlation

The processing protocol produces predictable particle sizes matched to appropriate injection devices. Particles measuring 1.0 mm can be injected through a 16-gauge needle or a 1.0-mm cannula; 0.5-mm particles can be injected through a 22-gauge needle or a 0.5-mm cannula; 0.25-mm particles can be injected through 23- to 25-gauge needles; and nanofat can be injected through 25- to 27-gauge needles.

To reduce trauma to fat grafts, the size of the harvested fat particles should correspond to the injection cannula or needle; however, cannulas used in lipoaspiration often lack clear size labels. Consequently, surgeons are often unaware of the optimal injection cannula size that would permit atraumatic injection of the fragile fat particles. This situation may result in adipocyte damage during injection and reduce the likelihood of survival at the recipient site [4].

Clinical application

The technique has been applied to breast reconstruction, breast augmentation, facial rejuvenation, revision breast reconstruction, primary breast reconstruction, and numerous other regenerative applications. Injection follows the standard principles of small-aliquot delivery in multiple tissue planes. The Micronizer has also been used in combination with platelet-rich fibrin (PRF) to enhance fat graft preparations, with PRF mixed into the micronized fat before injection to provide additional growth factors and a fibrin scaffold [8].

Technical observations

The direct cutting action is anticipated to generate less free oil than compressive techniques at equivalent particle sizes, suggesting reduced adipocyte rupture; however, formal quantification has not been performed. Processing time is comparable to that of traditional methods, requiring approximately 2-3 minutes per 20 mL of harvested fat for primary micronization. The device is economical, user-friendly, single-use, and disposable.

## Discussion

The Micronizer represents a conceptual departure from existing fat-processing devices by employing direct sharp dissection rather than compressive forces or indirect cutting. No adverse effects associated with the use of the device were noted. This distinction has potential implications for graft composition and viability.

Comparison with existing techniques

Several commercial systems are now available for mechanical fat micronization. Arcani R et al. conducted the first head-to-head comparison of eight commercially available nanofat-preparation devices and found significant variability in both technical performance and biological quality among the systems [9]. In that study, the Adinizer achieved the highest overall biological score and the greatest proportion of ASCs, while other devices demonstrated superior technical performance metrics. Microscopic analysis across all devices revealed preservation of adipocytes, vascular networks, and the extracellular matrix, challenging the assumption that emulsification or micronization completely disrupts tissue architecture.

Ma X et al. conducted a scoping review categorizing mechanical micronization techniques into shuffling-based emulsification, rotating blade systems, and filtration bag systems, highlighting the expanding landscape of processing options [7]. In a separate comparative study, Ma X et al. demonstrated that concentrated micronized fat processed using a spinning sharp blade achieved higher fat-retention rates and increased ASC concentrations compared with conventional microfat techniques, with reduced fibrosis and inflammation observed in vivo [10]. Langridge BJ et al. conducted a systematic review of fat-graft processing techniques and found that washing and filtration methods, including those involving commercial devices, resulted in superior long-term outcomes compared with centrifugation and decantation alone [11].

The Micronizer’s direct-cutting mechanism and tiered aperture system represent a distinct approach within this taxonomy. Unlike the Adinizer, in which the blade is oriented at 90° to the trajectory of fat passage, the Micronizer’s blade is aligned directly along the axis of particle movement. We hypothesize that direct cutting is less traumatic than indirect cutting because it minimizes lateral shearing forces on the tissue. Although direct comparative data between the Micronizer and other commercial devices are not yet available, the anticipated advantages of direct sharp dissection warrant further investigation.

Rationale for particle size reduction and cannula matching

The biological rationale for reducing fat particle size is well established. Eto H et al. demonstrated that only peripheral adipocytes located within 300 μm of the graft surface survive through direct plasmatic diffusion, with necrosis and regeneration occurring over a further 600 μm before revascularization takes place [3]. This three-zone model implies that particles 0.5-1.0 mm in diameter represent an optimal size range, as virtually all adipocytes within such particles fall within the surviving peripheral zone.

The relationship between particle size and the injection device is also clinically significant. James IB et al. demonstrated that injection cannulas of 14-gauge or larger diameter are more likely to create deposits with dimensions susceptible to central necrosis when injecting 1.0 mL per pass and recommended using smaller cannulas or lower volumes per pass [12]. The Micronizer’s stepwise processing protocol directly addresses this concern by enabling surgeons to tailor the particle size to the intended injection cannula or needle gauge, allowing particles to pass through the injection device with reduced compressive forces that could damage adipocytes during delivery.

Importance of preserving adipose tissue structure and viability

The preservation of adipose tissue architecture during processing is increasingly recognized as a critical determinant of graft quality. He Y et al. compared cotton-pad filtration, soft centrifugation (400 × g for one minute), and Coleman centrifugation (1,200 × g for three minutes) and found that, although volume-retention rates were similar across the groups, greater structural and functional damage from aggressive processing was associated with poorer graft quality, including decreased viability, reduced vessel density, lower vascular endothelial growth factor secretion, and increased vacuoles, necrotic areas, fibrosis, and inflammation [13]. These findings emphasize that adipocyte viability and tissue architecture, not merely stem-cell content, are crucial for high-quality graft outcomes.

Notably, Osinga R et al. demonstrated that mechanically shuffling lipoaspirated fat through interconnected syringes for up to 30 passes did not alter tissue viability or microscopic structure and had no significant effect on the SVF [14]. This finding is relevant to the Micronizer because it suggests that the intersyringe transfer component of the processing protocol is unlikely to be a major source of cellular damage. Rather, the mechanism of particle-size reduction, compressive emulsification versus sharp dissection, may be the more critical variable.

SVF concentration

**Figure 5:**
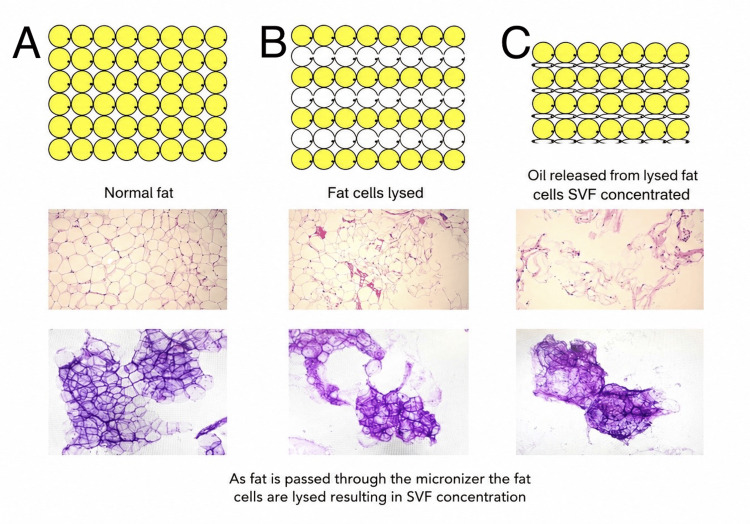
Schematic representation of SVF concentration during progressive micronization. (A) Normal fat with intact adipocytes. (B) Partial adipocyte lysis with preservation of the SVF. (C) Oil released from lysed adipocytes, resulting in relative concentration of the SVF. The lower panels show the corresponding H&E-stained histological sections. Scale bars were unavailable for these archival histological images. SVF: Stromal vascular fraction.

An additional anticipated benefit involves SVF concentration. As fat particles are reduced in size, some adipocytes are inevitably disrupted, releasing their lipid contents. Centrifugation removes this oil while retaining the SVF-rich connective-tissue matrix. The result is a concentrated preparation containing viable adipocytes and SVF components, including ASCs, endothelial cells, and pericytes (Figure 5). Previous studies have shown that enriching fat grafts with SVF has a significant effect on volume retention and viability [15]. A recent multicenter randomized controlled study by Wufuer M et al. demonstrated that SVF-enriched fat grafting achieved significantly higher fat-survival rates than conventional grafting at both six weeks (74.5% vs. 66.6%) and 24 weeks (71.3% vs. 62.0%) in facial fat transplantation [16].

Importantly, Becker H et al. demonstrated that harvesting with a 5-mm cannula yielded significantly more SVF than harvesting with a 1-mm cannula (mean: 0.23 cm³ vs. 0.11 cm³; p = 0.009) [17]. Furthermore, when 5-mm fat particles were resized to 1-mm particles using the Micronizer, the resulting mean SVF volume was 0.20 cm³, nearly double that obtained from fat harvested directly with a 1-mm cannula [17]. This finding provides direct evidence that harvesting with a larger cannula and subsequently micronizing the fat preserves a greater volume of SVF than harvesting with a smaller cannula, supporting the Micronizer’s role in a workflow that optimizes particle size while preserving SVF.

Microfat versus nanofat: distinct biological properties

The distinction between microfat and nanofat is not merely one of particle size; it reflects fundamentally different biological compositions and clinical applications. Yang Z et al. compared microfat, nanofat, and SVF gel and found that microfat maintained an intact histological structure with viable adipocytes, whereas nanofat contained no viable adipocytes or normal histological structure [18]. Microfat preserved the advantages of both subcutaneous volumetric restoration and skin-quality improvement, whereas the therapeutic potential of nanofat was primarily regenerative. The Micronizer’s tiered processing system allows surgeons to select the degree of processing appropriate for the clinical indication, preserving viable adipocytes for volumetric applications using microfat or producing a regenerative preparation using nanofat when skin rejuvenation is the primary goal.

Nanofat production

Traditional nanofat preparation, as described by Tonnard P et al., intentionally destroys adipocytes through emulsification to release ASCs for regenerative applications [5]. A recent expert consensus defined nanofat as requiring 20-30 passes through a 1.2-1.6-mm connector, followed by filtration [6]. The Micronizer can reduce fat to small particle sizes that permit injection in a manner similar to nanofat. Furthermore, SVF-rich tissue can be processed into ultrafine particles in a similar manner.

Limitations

This report has several important limitations. First, we present a technical description rather than comparative outcome data. Histological analyses comparing adipocyte viability in Micronizer-processed fat and fat processed using conventional devices would help evaluate the anticipated advantages. Second, long-term volume-retention data are not available. Third, particle sizes were estimated through gross visual comparison rather than formal microscopic analysis. Fourth, the observation that direct cutting generates less free oil than compressive techniques is based on qualitative assessment and has not been formally quantified. Fifth, there is a lack of quantitative validation and comparative studies.

Future directions

Rigorous evaluation of the Micronizer requires prospective comparative studies examining the following: (1) adipocyte viability using trypan blue exclusion or live/dead staining; (2) ASC concentration and function; (3) histological graft architecture; (4) long-term volume retention using volumetric MRI or 3D photography; (5) clinical outcomes across multiple applications, including breast and facial fat grafting; and (6) mechanical testing of shear forces. Direct comparison with other commercially available blade-equipped devices, including the Adinizer, would be particularly informative.

## Conclusions

The Micronizer offers a novel approach to fat graft processing by using direct sharp-blade technology to reduce particle size while minimizing anticipated adipocyte trauma. The device is designed to produce predictable particle sizes suitable for injection through fine cannulas and needles. Its dual-configuration design and direct-cutting mechanism distinguish it from other blade-equipped devices. The device offers an efficient, cost-effective, and easy-to-use method of reducing fat graft size with minimal anticipated compressive trauma, resulting in SVF-concentrated fat with presumed increased viability. Although preliminary clinical experience is encouraging, comparative studies evaluating cell viability and long-term outcomes are necessary to validate the anticipated advantages of this approach.
